# Outcomes of fertility investigations in a cohort of adults with perinatally acquired HIV-1 infection: a UK cross-sectional observational study

**DOI:** 10.1097/QAD.0000000000002745

**Published:** 2021-11-05

**Authors:** Thomas J. Pasvol, Jhia Teh, Danai Balfoussia, Rebecca Hall, Claire Petersen, Maryam Khan, Sara Ayres, Channa N. Jayasena, Caroline Foster, Sarah Fidler

**Affiliations:** a Clinical Trials Centre, Winston Churchill Wing, St Mary's Hospital, Imperial College London; b Imperial College Healthcare NHS Trust, London; c Research Department of Primary Care and Population Health, University College London; d Department of Andrology, Hammersmith Hospital & Section of Investigative Medicine, Imperial College London, UK.

## Abstract

There are no published studies of fertility measurements in people living with perinatally acquired HIV (PaHIV). We performed fertility investigations in 25 adults with PaHIV. Seven (78%) men had sperm morphology normal forms (%) below the fifth centile for the general population with four (44%) having no normal forms. Mean (SD) serum anti-Müllerian hormone level was 19.4 (9.5) pmol/l; lower than expected for this age group. A larger study is needed to verify our findings.

Adults with perinatally acquired HIV (PaHIV) have undergone puberty with a chronic viral infection, exposure to antiretroviral therapy (ART) and in some cases severe ill-health. We hypothesize that fertility in adults with PaHIV may be impaired.

We aimed to evaluate outcomes of fertility investigations in a cohort of adults with PaHIV.

This was a single-centre cross-sectional observational study. Patients aged 18 years or over with PaHIV attending a specialist HIV service in London, UK [1] between February 2018 and July 2019 were invited to participate.

Demographic information collected included age, ethnicity, BMI, nadir CD4^+^ count, smoking, alcohol and recreational drug use and past exposure to zidovudine (AZT), didanosine (DDI), stavudine (d4T) and efavirenz (EFV) (drugs previously shown to be associated with mitochondrial toxicity or sperm abnormalities [2–4]).

Blood was taken for lymphocyte subsets and plasma HIV viral load testing. An additional sample was taken from women for anti-Müllerian hormone (AMH) levels (a hormone produced by the granulosa cells of preantral and antral follicles, used as a biomarker of ovarian reserve). Female participants underwent transvaginal ultrasound (TVUSS) performed by a single gynaecologist at St Mary's Hospital, London to measure antral follicle count (AFC).

Male participants were given an appointment for semen production at Hammersmith Hospital, London. All samples were analysed according to WHO 2010 guidelines and UKNEQAS accreditation [5]. Samples were produced on site following 2–7 days of sexual abstinence, and incubated at 37 °C for liquefaction, up to 60 min prior to analysis. Sperm morphology was analysed on Papanicolaou prestained slides, using Kruger strict criteria.

For men, lower reference limits for seminal fluid analysis (SFA) parameters were taken from one-sided fifth centile values from the general population of young men applying to donate sperm samples for trials of hormonal contraception (pooled results from Australia, UK, USA, Chile and Germany) [5].

Descriptive characteristics were summarized using numbers and percentages for categorical variables, medians and interquartile ranges (IQR) for nonnormally distributed numerical variables and means and standard deviations (SD) for normally distributed variables.

Ethical approval was obtained from Camden & Kings Cross Research Ethics Committee (REC) on 18/07/2017. IRAS project ID: 231804. REC reference: 17/LO/1742. Sponsor: Imperial College.

Twenty-one women and 15 men with PaHIV were enrolled, of whom 16 (76%) women and 9 (60%) men completed the study. All patients were receiving ART, with 15/16 (94%) women and 7/9 (78%) men having HIV viral load less than 50 copies/ml at the study visit. Two of 16 (12%) women and no men had a CD4^+^ count less than 200 cells/μl (Table 1).

**Table 1:** Demographics and results of fertility investigations for participants who completed the study.

	Women (*n* = 16)	Men (*n* = 9)
Age (years) (mean ± SD)	24 ± 4	27 ± 4
African/Caribbean/mixed race, *n* (%)	14 (88%)	8 (89%)
BMI (kg/m^2^)	22.8 (21.2–24.9)	25.8 (20.9–27.0)
Nadir CD4^+^ count (cells/μl)	190 (94–263)	300 (180–450)
CD4^+^ cell count at time of study (cells/μl)	693 (474–1114)	740 (360–883)
CD4^+^:CD8^+^ ratio at time of study	0.9 (0.6–1.1)	0.9 (0.5–1.3)
HIV viral load <50 copies/ml at time of study [*n* (%)]	15 (94%)	7 (78%)
Current cigarette smoking [*n* (%)]	4 (25%)	2 (22%)
Current regular alcohol intake [*n* (%)]	10 (63%)	6 (67%)
Current recreational drug use [*n* (%)]	0 (0%)	3 (33%)
Known exposure to AZT, DDI, d4T^a^ [*n* (%)	3 (19%)	6 (67%)
Known exposure to EFV^a^, [*n* (%)]	4 (25%)	2 (22%)
Female fertility investigations		
AMH (pmol/l) [mean ± SD]	19.4 ± 9.5	
AFC [mean ± SD]	23 ± 9	

Male fertility investigations		
	Median (IQR)	Below fifth centile for the general population^b^ (*n*)
Sperm volume (ml)	3.1 (1.6–1.9)	0 (0%)
Sperm concentration (10^6^/ml)	51 (17–75)	2 (22%)
Total number (10^6^/ejaculate)	105 (70–264)	2 (22%)
Total motility (PR + NP, %)	57 (33–66)	3 (33%)
Progressive motility (PR, %)	47 (29–52)	3 (33%)
Normal forms (%)	2 (0–4)	7 (78%)

Data are presented as median (IQR) unless otherwise indicated. AFC, antral follicle count; AMH, anti-Mullerian hormone; BMI, body mass index; IQR, interquartile range; SD, standard deviation; PR, progressive motility (WHO, 1999 grades a + b); NP, nonprogressive motility (WHO, 1999 grade c).

a Drugs that have been shown to be associated with mitochondrial toxicity or sperm abnormalities.

b Distribution of lower reference limits was selected as the one-sided fifth centile for semen variables from the general population of unscreened men [16].

Of the 16 (76%) women who completed the study, mean (SD) age was 24 (4) years and all were nulliparous. Mean (SD) AMH for the study group was 19.4 (9.5) pmol/l. No participants had ‘very low’ AMH levels (<4.3 pmol/l). Mean (SD) AFC was 22.6 (9.8).

Mean (SD) age of the male participants was 27 (4) years and none reported fathering children. Eight (89%) men had SFA results below the 5th centile for at least one parameter compared to the general population. Seven (78%) males had sperm morphology normal forms (%) below the 5th centile with four (44%) having no normal forms (Table 1).

This is the first study to report outcomes of fertility investigations in a cohort of adults with PaHIV. Although this is a small study, we describe abnormal SFA results for at least one parameter amongst 89% of young men tested and lower mean serum AMH than expected for women of this age.

Mean (SD) AMH in our study was 19.4 (9.5) pmol/l compared to 21.4–23.0 pmol/l found in cohorts of young women with horizontally acquired HIV [6,7] and 25–30 pmol/l in HIV-uninfected women [8].

Compared with the AFC age-related normogram produced by McGill University [9], the mean AFC for our population was at the 97th centile for their age group – indicating good ovarian reserve. However, these reference data were derived from women who have been diagnosed with infertility and other normograms demonstrate a wide variation in results [10,11].

We observed that 89% of young PaHIV men had abnormal SFA for at least one parameter, compared to 19% reported in a Spanish cohort of 139 men with horizontally acquired HIV [2]. The most striking observation from our SFA results was the higher than expected prevalence of teratospermia (a reduced percentage of morphologically normal spermatozoa), an established marker of male factor infertility [5]. Teratospermia has been reported in men with horizontally acquired HIV [12–14], although not to such a degree as in our small PaHIV cohort.

A strength of this study is the originality of the work. No previous study has explored outcomes of fertility investigations in adults with PaHIV. Although a number of studies have looked at pregnancy or conception outcomes in PaHIV, participants in this study were not selected on the basis that they were actively trying to conceive. Therefore, it could be argued that our results may be more generalizable to people living with PaHIV overall.

A major limitation of this study is the small sample size; recruitment was limited by the personal nature of the tests. Our study would have benefitted from longitudinal data, including serial measurements of SFA and an appropriate control group. Importantly, fertility investigations are mainly used in the context of assisted conception and it is unclear whether they can predict reproductive potential.

We propose three potential mechanisms for reduced reproductive fitness in this group: chronic ill health and inflammation; ART toxicity; lifestyle factors, such as smoking, cannabis use and obesity. Reassuringly, healthy live births are increasingly reported in perinatal cohorts [15].

This is the first study to report teratospermia in men with PaHIV. Although mean serum AMH was lower than expected, our sample size was too small to conclude that ovarian reserve may be impaired. A larger study with an age and ethnically matched control group is needed.

## Acknowledgements

Financial support: This study was co-funded by a grant from Imperial Health Charity (reference SGF17_144) and the NIHR Imperial Biomedical Research Centre (BRC). C.N.J is funded by an NIHR post-doctoral fellowship. S.F. is supported by the NIHR Imperial BRC. The views expressed are those of the authors and not necessarily those of Imperial Health Charity, NIHR or The Department of Health and Social Care.

Author contributions: T.J.P. -- study lead, conception and design of the study and all study documents, acquisition of data, analysis and interpretation of data, drafting the article, final approval of the submitted manuscript. J.T. -- aided conception and design of the study, designing study database and collected results, revising the article critically for important intellectual content, final approval of the submitted manuscript. D.B. -- conception and design of the study, performed all ultrasound scans, analysis and interpretation of data, revising the article critically for important intellectual content, final approval of the submitted manuscript. R.H. – lead nurse for the study, saw patients at each visit, revising the article critically for important intellectual content, final approval of the submitted manuscript. C.P. – enrolling and consenting patients, booking follow-up visits, revising the article critically for important intellectual content, final approval of the submitted manuscript. M.K. – laboratory analysis, revising the article critically for important intellectual content, final approval of the submitted manuscript. S.A. – recruiting to the study, acquisition of data revising the article critically for important intellectual content, final approval of the submitted manuscript. C.N.J. -- conception and design of the study, interpretation of data in particular semen analysis results, revising the article critically for important intellectual content, final approval of the submitted manuscript. C.F. -- conception and design of the study, enrolling patients, interpretation of data, revising the article critically for important intellectual content, final approval of the submitted manuscript. S.F. – primary investigator, the conception and design of the study, acquisition of data, or analysis and interpretation of data, revising the article critically for important intellectual content, final approval of the submitted manuscript.

### Conflicts of interest

There are no conflicts of interest.
